# Significance of Early Postoperative Magnetic Resonance Imaging following Intracranial Meningioma Resection

**DOI:** 10.3390/jcm12144733

**Published:** 2023-07-17

**Authors:** Mizuho Inoue, Masaya Miyazaki, Soichi Oya

**Affiliations:** 1 Department of Neurosurgery, Saitama Medical Center, Saitama Medical University, Saitama 350-8550, Japan; zumi.eunoi@gmail.com; 2 Department of Radiology, Saitama Medical Center, Saitama Medical University, Saitama 350-8550, Japan; mmiyazak.smc@gmail.com

**Keywords:** meningioma, MRI, postoperative

## Abstract

The significance of early postoperative magnetic resonance imaging (MRI) for meningioma resection has not yet been evaluated. We retrospectively reviewed patients with intracranial meningiomas resected at our institute between 2011 and 2021. Early postoperative MRI with contrast enhancement was routinely performed within 48 h after surgery while first follow-up MRI was performed approximately after 6 months. MRI findings were reviewed, and the risk factors for postoperative infarction and early recurrence were analyzed. Among the 245 resections performed, early postoperative MRI was performed in 200 cases. Postoperative radiological and symptomatic infarctions occurred in 54 (27%) and 17 patients (9%), respectively. Diameter > 5 cm (*p* = 0.015) and skull base location (*p* = 0.010) were independent risk factors for radiological infarctions. Follow-up postoperative MRI performed in 180 patients (90%) detected early recurrence in 24 patients (13%). Non-gross total resection was an independent risk factor for early recurrence (*p* < 0.0001). Additionally, early recurrence after gross total resection occurred significantly more frequently in meningiomas with dural sinus involvement than in those without (8.3% vs. 0%, *p* = 0.018). Thus, early postoperative MRI may enable the timely assessment of postoperative neurological deficits, especially after large skull base meningioma resections along with accurate detection of early recurrence, which is critical for meningiomas with dural sinus involvement.

## 1. Introduction

Early postoperative imaging after brain tumor resection is generally performed during the hospital stay, either in the form of computed tomography (CT) or magnetic resonance imaging (MRI). The significance of postoperative imaging studies after intracranial tumor resection may vary among different tumor types. For instance, early postoperative MRI with contrast enhancement within 72 h after surgery is recommended for high grade gliomas to better distinguish residual tumors from postoperative changes [1]. Early postoperative MRI is also beneficial for the detection of ischemic changes on diffusion-weighted imaging (DWI) in case of neurological deficit [2]. 

However, for meningiomas, the most common benign brain tumor, the significance of early postoperative MRI has not yet been elucidated. Early postoperative MRI after meningioma resection may contribute to the improvement of acute postoperative care by early detection of unexpected vascular events or brain swellings and accurate evaluation of recurrence by comparing early and follow-up postoperative MRIs. In contrast, the indication of postoperative MRI should be carefully considered because it requires a longer scan time than CT scanning and therefore could pose a risk, especially in patients with unstable vital signs. We routinely implement a protocol of MRI within 48 h of meningioma surgery whenever possible. In this study, we retrospectively reviewed meningioma surgeries in our institute to evaluate the significance of early postoperative MRI after meningioma resection in the modern neurosurgical era.

## 2. Materials and Methods

### 2.1. Patient Enrollment and Protocol for MRI

We retrospectively reviewed all resected meningioma cases between 2011 and 2021 at our institute. A total of 245 meningioma resections were performed during the study period. Our routine protocol for postoperative imaging studies included immediate postoperative CT before admission to the ICU, and early postoperative MRI within 48 h after surgery on postoperative day (POD) 1 or 2. MRI was not performed if contraindicated due to the use of pacemaker or other implanted devices. In addition, MRI was postponed if the patient’s vital signs were unstable 48 h after surgery or when the day after surgery was followed by holidays lasting more than two days.

Protocols for early postoperative MRI (<48 h) included diffusion-weighted imaging (DWI), T1-weighted imaging (T1WI), T2-weighted imaging (T2WI), fluid attenuated inversion recovery (FLAIR) imaging, susceptibility-weighted imaging (SWI), and T1WI with gadolinium (Gd) enhancement. MR angiography or venography was included in the protocol when arterial or venous patency was suspected. For emergency situations such as severe brain edema, adequate treatment was provided. The interpretation of MRI findings was performed and agreed upon by both the neurosurgeons and the radiologists. After discharge, the patients were followed up in the outpatient setting, and a second postoperative MRI was performed approximately six months after surgery. Subsequently, periodic MRI scans were obtained approximately every 6–12 months until the final outpatient follow-up.

### 2.2. Data Collection

Cases that matched the inclusion criteria were reviewed for the following patient and tumor characteristics: age, sex, initial or re-do surgery, history of prior radiation, preoperative symptoms, maximum diameter, location (skull base/non-skull base), involvement of the dural sinuses (superior sagittal, transverse, sigmoid, straight, or cavernous sinus), presence of peritumoral brain edema (PTBE), and WHO grade. Convexity, parasagittal, falx, or ventricular meningiomas were classified as non-skull base meningiomas, whereas other meningiomas arising from the base of the skull were considered skull base meningiomas. The WHO grade was determined based on the 2016 edition of the WHO Classification of Tumors of the Central Nervous System [3]. For the tumors resected before WHO 2016 was adopted, we re-assessed the grade according to WHO 2016 grading system. Information on the incidence of postoperative infarction, contusion, patency of related dural sinuses, and presence of recognizable residual tumors was collected from early postoperative MRI. Gross total resection (GTR) was defined as no appreciable residue on postoperative MRI. Subtotal resection (STR) was defined as a small but apparent residual tumor, and partial resection (PR) was defined as a resection rate of less than 70%.

**Figure 1:**
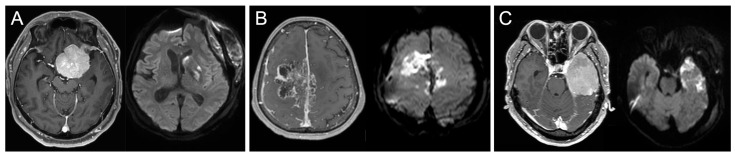
Postoperative DWI signal changes. Paired preoperative T1Gd and postoperative DWI are shown to demonstrate different patterns of postoperative DWI signal changes, including arterial (**A**) or venous (**B**) infarction or contusion (**C**).

Regarding the definition of postoperative infarctions, bright signal intensity areas on DWI with decreased apparent diffusion coefficient values were interpreted as infarctions if they were related to the arterial (Figure 1A) or venous territories (Figure 1B). Arterial infarctions included major arterial territory, cortical artery, and perforator infarctions. A thin, high-intensity DWI rim along the resection cavity that did not match the arterial territory was interpreted as a contusion (Figure 1C). The infarctions were classified as either symptomatic or asymptomatic. Ischemic changes were considered symptomatic if they were associated with new neurological symptoms or worsening of preoperative symptoms corresponding to their location. 

For patients who underwent follow-up imaging studies, postoperative MRI obtained 6 months after surgery was compared with early postoperative MRI to evaluate the role of early MRI in detecting recurrence. 

### 2.3. Statistical Analysis

Demographic data were assembled and analyzed to identify factors associated with postoperative infarction and early recurrence. For univariate analysis, binary variables were compared using Pearson’s chi-square test. We included all variables from the univariate analysis in multivariate logistic regression to identify independent risk factors. Statistical significance was set at *p* < 0.05. All statistical analyses were performed using the JMP software (version 14.0; SAS Institute Inc., Cary, NC, USA).

## 3. Results

### 3.1. Patient and Tumor Characteristics

**Figure 2:**
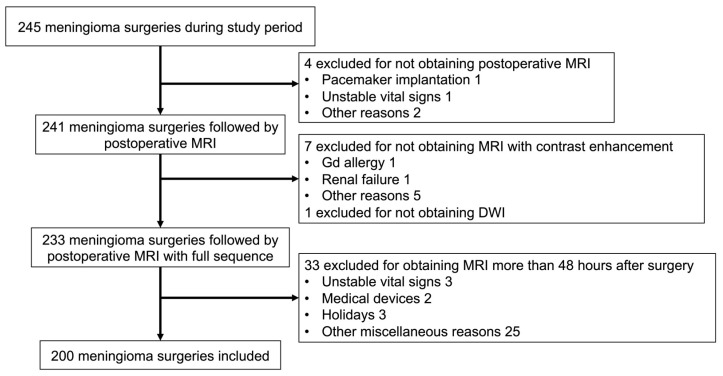
Flowchart demonstrating the inclusion and exclusion criteria of the present study.

A total of 245 meningioma surgeries were performed during the study period. A flowchart showing the inclusion and exclusion criteria is illustrated in Figure 2. Among the 245 meningioma resections, postoperative MRI was not conducted in four cases. Eight patients were excluded due to the lack of contrast MRI or DWI sequences. Among the remaining 233 surgeries, 33 were excluded in which postoperative MRI was performed > 48 h after surgery. In total, 200 surgeries involving 185 patients were included in this study. The median length of follow-up is 3.13 [interquartile range 1.23–5.50] years.

The mean age of the patients was 61.7 years (SD 12.8), and 129 (65%) were women (Table 1). This study included 174 initial surgeries and 26 re-do surgeries (two patients having four surgeries and nine having two surgeries). In 17 (9%) patients, the tumor was previously treated with radiation, including stereotactic radiosurgery. The mean maximum tumor diameter was 4.1 cm (SD, 1.6). The tumors were located in the skull base region in 124 patients (62%). The dural sinuses were associated with tumors in 114 patients (57%). PTBE was observed in 107 patients (54%). There were 167 WHO grade I meningiomas (84%), 28 atypical meningiomas (14%), and five anaplastic meningiomas (3%). GTR was achieved in 134 (67%) patients. Two surgeries were performed using the transsphenoidal approach, whereas the remaining were performed via craniotomy.

### 3.2. Early Postoperative MRI Findings and Risk Factors for Postoperative Infarction

A summary of the early findings is presented in Table 2. The overall incidence of postoperative infarction was 27% (54/200), including symptomatic infarctions in 17 patients (9%). Among them, 50 (92.6%) had arterial infarctions and four had venous infarctions. One patient with an arterial infarction subsequently required a decompressive craniectomy. Two patients with venous infarction were treated with osmotic diuretics and steroids. The remaining patients did not require additional treatment for infarction. Sinus abnormalities were found in 18 patients (9%), including sinus thrombosis in seven patients (4%) and sinus stenosis in 11 patients (6%). None of the patients were symptomatic; however, one patient with the superior sagittal sinus stenosis was treated with prophylactic anticoagulant therapy to prevent subsequent thrombosis. Contusion around the tumor was identified on either T2, FLAIR, DWI, or SWI in 130 patients (65%). Residual tumors were detected on early postoperative MRI in 66 patients (33%). Adjuvant radiation was administered to six patients (one patient with grade I meningioma, three with grade II meningioma, and two with grade III meningioma) before follow-up MRI 6 months after surgery. Another patient with grade III meningioma was treated with re-do surgery within 6 months of the initial surgery.

We investigated the risk factors for postoperative infarction (Table 3). On univariate analysis, the presence of preoperative symptoms (*p* = 0.017), maximum diameter > 5 cm (*p* = 0.004), skull base location (*p* = 0.032), non-GTR (*p* = 0.015), and WHO grade II or III (*p* = 0.009) were significantly associated with postoperative radiological infarction. Tumor size (*p* = 0.008) and WHO grade II or III (*p* = 0.029) were also significantly associated with postoperative symptomatic infarctions. In multivariate analysis, a maximum diameter > 5 cm (odds ratio [OR] 2.56; 95% confidence interval [CI] 1.20–5.44; *p* = 0.015) and skull base location (OR 3.03; 95% CI 1.31–7.03; *p* = 0.010) were independent risk factors for postoperative radiological infarction. No independent risk factors of symptomatic infarction were identified.

### 3.3. Late Postoperative MRI Findings and Risk Factors for Early Recurrence

Late postoperative MRI findings were obtained at the 6-month follow-up for 180 patients (90%). Recurrence or interval growth of the residual portions was observed in 24 cases (13%), including 19 grade I and five grade II meningiomas. Fourteen (8%) patients required salvage treatment. Among them, eight grade I and four grade II meningiomas were treated with radiation therapy. One patient with grade I meningioma and one with grade II were treated with re-do surgeries.

**Figure 3:**
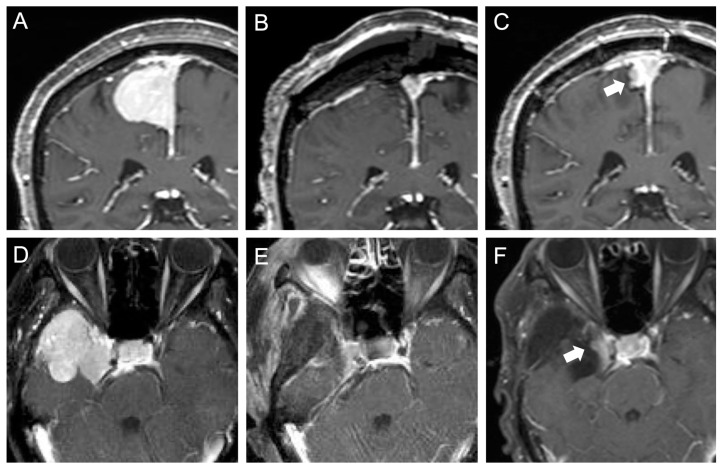
Demonstrative cases of a non-skull base (**A**–**C**) and a skull base (**D**–**F**) meningioma with dural sinus involvement showing early recurrence after gross total resection. (**A**) Preoperative T1Gd coronal image shows parasagittal meningioma involving superior sagittal sinus. (**B**) Early postoperative MRI on POD1 shows no apparent residuals. (**C**) Follow-up MRI 6 months after surgery shows the recurrent tumor adjacent to the superior sagittal sinus (arrow). The patient was closely followed with repeated MRI in another 6 months, which showed further growth of the recurrent tumor. Stereotactic radiation therapy was administered and the tumor regressed without any regrowth for 3 years. (**D**) Preoperative T1 Gd axial image shows right medial sphenoidal ridge meningioma involving cavernous sinus. (**E**) Early postoperative MRI on POD1 shows no apparent residuals. (**F**) Follow-up MRI 7.5 months after surgery shows the recurrent tumor at the lateral wall of the cavernous sinus (arrow). Gamma knife surgery was performed and the tumor size was stable for 4.5 years, after which it showed regrowth and required surgical resection.

We also investigated risk factors for early recurrence within 6 months (Table 4). In univariate analysis, redo surgeries (*p* = 0.029), sinus involvement (*p* = 0.030), and non-GTR (*p* < 0.0001) were significantly associated with recurrence 6 months postoperatively. In multivariate analysis, non-GTR was the only independent risk factor for early recurrence (OR 11.4; 95% CI 3.54–36.0; *p* < 0.0001). We further investigated the significance of postoperative MRI findings in patients with dural sinus involvement. Among 124 GTR cases with the completion of a 6-month follow-up, early recurrence was confirmed in five cases (4.0%) on MRI 6 months later, and all tumors had dural sinus involvement, resulting in a significantly higher recurrence rate in tumors with dural sinus involvement than in tumors without (5/60 [8.3%] vs. 0/64 [0%]; *p* = 0.018). Although dural sinus involvement was significantly more frequent in skull base meningiomas compared with non-skull base meningiomas (78/124 [63%] vs. 36/76 [47%]; *p* = 0.031), early recurrences after GTR were equally confirmed in both skull base and non-skull base meningiomas with dural sinus involvement (3/36 [8.3%] vs. 2/24 [8.3%]; *p* = 1.00). Two illustrative cases are shown in Figure 3.

## 4. Discussion

### 4.1. Significance of the Early Postoperative MRI in Brain Tumor Surgery

It is standard practice to evaluate the extent of resection after brain tumor resection using imaging. Although CT can show the approximate extent of resection, MRI allows for a more accurate evaluation. In addition, MRI provides additional information, such as ischemic changes and contusions associated with resection. However, MRI in the immediate postoperative period poses a risk of worsening the patient’s condition if the vital signs are unstable. In addition, transporting patients who cannot move independently with several medical staff members may be a burden. Therefore, clarifying the significance of MRI will help identify patients who should undergo early MRI, and thus improve the efficiency of postoperative management. Several studies have assessed the significance of early postoperative MRI for brain tumors. For high-grade gliomas, early postoperative MRI with contrast enhancement within 72 h after surgery is recommended to better distinguish residual tumors from postoperative changes [1]. For metastatic tumors, early postoperative MRI is beneficial for detecting unexpected residual tumor [4] and postoperative ischemic lesions [5]. The significance of immediate postoperative MRI is not well established for pituitary adenomas compared to gliomas and metastatic tumors; however, a single center study showed that early postoperative MRI had a greater sensitivity for detecting residual tumor than delayed postoperative MRI [6]. Although meningioma is the most common brain tumor [7], the significance of early postoperative MRI after meningioma resection has not been investigated in depth. Since 2011, our institution has routinely performed early MRI within 48 h after surgery. Therefore, we aimed to delineate the role of early postoperative MRI during the acute postoperative period.

### 4.2. Role of Early Postoperative MRI to Detect Ischemia after Meningioma Resection

Most previous studies have focused on the significance of early postoperative MRI in glioma resection. These studies revealed that postoperative infarctions are more often seen in patients with new deficits after resection [8] and that infarctions detected on DWI findings within 72 h after brain tumor resection were associated with poor recovery from new postoperative neurological deficits [9]. These findings highlight the importance of evaluating postoperative acute ischemia after glioma resection. The incidence of radiological infarction following glioma or metastatic brain tumor surgery is reportedly 26–44%, and the incidence of symptomatic infarction is 6–13% [2,5,10,11]. Regarding meningiomas, a review of 125 convexity meningioma resections showed that postoperative DWI obtained 24–48 h after surgery demonstrated restriction > 1 cm in 22% of the cases and symptomatic infarction in 11% [12]. Another study reported that early MRI performed within 72 h after meningioma resection detected peritumoral infarction in 34.9% of patients [13]. Our results are consistent with these studies. In addition, these two studies emphasized that permanent neurological deficits were more common in patients with infarctions on early postoperative MRI. Therefore, early MRI evaluation of infarction is recommended as a routine examination to assess the prognosis of postoperative neurological deficits in meningiomas as well as intra-axial tumors. A previous study critically discussed the need for early postoperative imaging in asymptomatic patients who underwent resection of meningioma in easily accessible locations [14]. However, patients with deep skull base meningiomas were not included in their study, and CT was used instead of MRI in two-thirds of patients. In contrast, in the present study, 62% of patients had skull base meningiomas, and all patients underwent early postoperative MRI. Therefore, we believe that a direct comparison of our results with this previous study is difficult. If, for various hospital reasons, MRI cannot be performed immediately after surgery for all patients, it would be helpful to know which characteristics of meningiomas warrant an early assessment of postoperative infarction. The multivariate analysis revealed that large tumors with a maximum diameter of >5 cm and skull base location were independent risk factors for radiological infarction. The increased risk of larger meningiomas could be because larger tumors are more likely to involve surrounding arteries or veins. Although none of the factors assessed in this study were significantly associated with an increased risk of symptomatic infarction in the multivariate analysis, patients with tumor size > 5 cm showed a trend toward a higher risk of symptomatic infarction. Patients with large skull base meningiomas may present with disturbed consciousness after surgery and may require differentiation from epilepsy, infarction, or impaired venous perfusion. Our results suggest that immediate postoperative MRI may be beneficial in such cases. 

### 4.3. Role of Early Postoperative MRI to Evaluate Recurrence of Meningiomas

Multivariate analysis demonstrated that non-GTR was a risk factor for recurrence at 6 months follow-up. A previous study showed that residual tumor volume is associated with the postoperative annual growth rate; in particular, whether the volume of the residual tumor is 5 cm^3^ or less produces the most significant difference in the annual growth rate [15]. The extent of meningioma resection has been evaluated using the Simpson grading system. However, this system is based on the subjective judgment of the surgeon and is not exempt from inter- and intra-observer biases [16]. In contrast, an integrated assessment combining genetic and molecular analyses with the extent of resection allows for a more accurate prediction of recurrence [17]. Accordingly, the extent of resection should be assessed only when postoperative Gd enhancement does not affect the interpretation of the enhanced area. Thus, early evaluation using MRI may contribute to an accurate prediction of recurrence risk.

The effectiveness of early postoperative DWI has been demonstrated in distinguishing residual tumors from contrast-enhanced ischemic lesions in gliomas [18]. In contrast, in meningiomas, which are extra-axial tumors with a clear boundary with the brain parenchyma, the border with surrounding tissue other than the brain parenchyma is critical for assessing the degree of resection and detecting recurrence. For example, it may be difficult to distinguish between postoperative changes and recurrence in tumors that contact or invade the dural or cavernous sinus, tumors that invade other large cortical veins, and skull base tumors that invade the extracranial muscle or soft tissue. In our study, early recurrence after GTR at the 6-month follow-up occurred only in meningiomas with dural sinus involvement, indicating the importance of accurate radiological evaluation of residual tumors in the vicinity of the dural sinus. The boundary between the tumor and dural sinus is difficult to discern on imaging [19]. In addition, although various hemostats are frequently used to control bleeding from the dural sinus wall, they may change delineation on contrast MRI over time, which may affect the interpretation of the findings [20]. Given the characteristics of meningioma resection, it is useful to perform early postoperative MRI for precise baseline comparisons.

### 4.4. Study Limitations

This study had a few limitations. First, although we attempted routine implementation of MRI within 48 h, we could not obtain an MRI within 48 h of the procedure in 18% of the surgeries. Therefore, we cannot conclude that MRI should be performed within 48 h in all cases. We believe that the safety management systems of each hospital should be taken into consideration. Second, we analyzed MRI findings within 48 h after surgery, but most studies on the significance of early MRI in other brain tumors dealt with MRI findings within 72 h. From the present analysis, it is unclear whether this benefit can be observed even with MRIs performed a few more days later. However, we recommend that MRI be performed within 48 h if patients exhibit worsening preoperative symptoms or new neurological deficits. Third, the incidence of postoperative infarction depends on the definition of DWI abnormalities. We followed previous reports and excluded thin DWI high-signal rims along the resection cavity, which indicate a minor contusion in the tumor cavity [2,5,11,13]. We believe that our method is reasonable because the incidence of postoperative infarction in this study was similar to that previously reported.

## 5. Conclusions

Based on our results, a maximum diameter > 5 cm and skull base location are independent risk factors for postoperative radiological infarctions. Early postoperative MRI may allow the timely assessment of postoperative neurological deficits, especially after surgery for large skull base meningiomas. While non-GTR is associated with an increased risk of early recurrence, and early recurrence of GTR is more frequently observed in meningiomas involving the dural sinus. Given that meningiomas often arise near the dural sinus, early postoperative MRI is considered an effective baseline imaging to accurately assess recurrence.

## Figures and Tables

**Table 1:** Patient and tumor characteristics.

**Patient Characteristics (n = 200)**		
Mean age (years)		61.7 (SD 12.8)
Sex	Male	71 (36%)
	Female	129 (65%)
Initial or Re-do	Initial	174 (87%)
	Re-do	26 (13%)
Prior radiation		17 (9%)
Pre-op symptoms	Symptomatic	159 (80%)
	Asymptomatic	41 (21%)
**Tumor Characteristics (n = 200)**		
Mean maximum diameter (cm)		4.1 (SD 1.6)
Location	Skull base	124 (62%)
	Non skull base	76 (38%)
Dural Sinus involvement	Involved	114 (57%)
	Not involved	86 (43%)
PTBE	Present	107 (54%)
	Absent	93 (47%)
WHO grade	I	167 (84%)
	II	28 (14%)
	III	5 (3%)
Extent of resection	GTR	134 (67%)
	STR or PR	66 (33%)

**Table 2:** Early postoperative MRI findings.

Early Postoperative MRI Findings (n = 200)		
Infarction	Overall	54 (27%)
	Symptomatic	17 (9%)
Contusion		130 (65%)
Dural sinus problems	Thrombosis	7 (4%)
	Stenosis	11 (6%)
Residual on MRI		66 (33%)
Additional treatment for residual		7 (4%)

**Table 3:** Risk factors of postoperative infarction.

Factor	Radiological Infarction					Symptomatic Infarction				
	Infarction (n = 54)	No Infarction (n = 146)	*p* Value, Univariate	*p* Value, Multivariate	OR (95% CI)	Infarction (n = 17)	No Infarction (n = 183)	*p* Value, Univariate	*p* Value, Multivariate	OR (95% CI)
Age > 60 years	37 (69%)	83 (57%)	0.13	0.13	1.77 (0.84–3.72)	8 (47%)	112 (61%)	0.25	0.11	0.40 (0.13–1.24)
Male sex	23 (43%)	48 (33%)	0.20	0.34	1.43 (0.69–2.94)	9 (53%)	62 (34%)	0.12	0.34	1.70 (0.58–5.00)
Redo surgeries	10 (19%)	16 (11%)	0.16	0.89	0.91 (0.22–3.66)	2 (12%)	24 (13%)	0.87	0.44	0.30 (0.01–6.26)
Prior radiation	8 (15%)	9 (6%)	0.051	0.53	1.68 (0.33–8.45)	2 (12%)	15 (8%)	0.61	0.50	2.86 (0.13–60.8)
Pre-op symptoms	49 (91%)	110 (75%)	0.017	0.098	2.60 (0.84–8.06)	16 (94%)	143 (78%)	0.12	0.27	3.47 (0.39–31.1)
Size > 5 cm	22 (41%)	30 (21%)	0.004	0.015	2.56 (1.20–5.44)	9 (53%)	43 (24%)	0.008	0.063	2.85 (0.95–8.57)
Skull base location	40 (74%)	84 (58%)	0.032	0.010	3.03 (1.31–7.03)	10 (59%)	114 (62%)	0.78	0.89	0.92 (0.28–3.05)
PTBE	33 (61%)	74 (51%)	0.19	0.51	1.29 (0.61–2.71)	12 (71%)	95 (52%)	0.14	0.37	1.78 (0.51–6.24)
Dural sinus involvement	33 (61%)	81 (55%)	0.48	0.83	0.92 (0.44–1.95)	10 (59%)	104 (57%)	0.87	0.56	0.70 (0.21–2.33)
Non-GTR	25 (46%)	41 (28%)	0.015	0.50	1.29 (0.62–2.69)	9 (53%)	57 (31%)	0.068	0.15	2.32 (0.73–7.37)
WHO grade II or III	15 (28%)	18 (12%)	0.009	0.098	2.27 (0.86–6.01)	6 (35%)	27 (15%)	0.029	0.28	2.06 (0.56–7.61)

OR = odds ratio.

**Table 4:** Risk factors for early recurrence on the 6-month follow-up MR.

Factor	Recurrence (n = 24)	No Recurrence (n = 156)	*p* Value, Univariate	*p* Value, Multivariate	OR (95% CI)
Age > 60 years	16 (67%)	91 (58%)	0.44	0.99	1.00 (0.34–2.92)
Male sex	10 (42%)	53 (34%)	0.46	0.56	1.36 (0.49–3.79)
Redo surgeries	6 (25%)	15 (10%)	0.029	0.18	3.34 (0.58–19.1)
Prior radiation	3 (13%)	9 (6%)	0.22	0.47	0.45 (0.05–4.02)
Size > 5 cm	9 (38%)	36 (23%)	0.13	0.41	1.58 (0.54–4.62)
Skull base location	16 (67%)	94 (60%)	0.55	0.57	0.72 (0.23–2.23)
Dural sinus involvement	18 (75%)	81 (52%)	0.03	0.38	1.66 (0.54–5.08)
Non-GTR	19 (79%)	37 (24%)	<0.0001	<0.0001	11.4 (3.64–36.0)
WHO grade II or III	5 (21%)	22 (14%)	0.39	0.67	0.74 (0.19–2.90)

OR = odds ratio.

## Data Availability

The data presented in this study are available upon request from the corresponding author.

## References

[1] Albert F.K., Forsting M., Sartor K., Adams H.P., Kunze S. (1994). Early postoperative magnetic resonance imaging after resection of malignant glioma: Objective evaluation of residual tumor and its influence on regrowth and prognosis. Neurosurgery.

[2] Gempt J., Förschler A., Buchmann N., Pape H., Ryang Y.M., Krieg S.M., Zimmer C., Meyer B., Ringel F. (2013). Postoperative ischemic changes following resection of newly diagnosed and recurrent gliomas and their clinical relevance. J. Neurosurg..

[3] Louis D.N., Perry A., Reifenberger G., von Deimling A., Figarella-Branger D., Cavenee W.K., Ohgaki H., Wiestler O.D., Kleihues P., Ellison D.W. (2016). The 2016 World Health Organization classification of tumors of the central nervous system: A summary. Acta Neuropathol..

[4] Olesrud I.C., Schulz M.K., Marcovic L., Kristensen B.W., Pedersen C.B., Kristiansen C., Poulsen F.R. (2019). Early postoperative MRI after resection of brain metastases-complete tumour resection associated with prolonged survival. Acta Neurochir..

[5] Gempt J., Gerhardt J., Toth V., Hüttinger S., Ryang Y.M., Wostrack M., Krieg S.M., Meyer B., Förschler A., Ringel F. (2013). Postoperative ischemic changes following brain metastasis resection as measured by diffusion-weighted magnetic resonance imaging. J. Neurosurg..

[6] Alhilali L.M., Little A.S., Yuen K.C.J., Lee J., Ho T.K., Fakhran S., White W.L. (2020). Early postoperative MRI and detection of residual adenoma after transsphenoidal pituitary surgery. J. Neurosurg..

[7] Ostrom Q.T., Price M., Neff C., Cioffi G., Waite K.A., Kruchko C., Barnholtz-Sloan J.S. (2022). CBTRUS statistical report: Primary brain and other central nervous system tumors diagnosed in the United States in 2015–2019. Neuro Oncol..

[8] Jakola A.S., Berntsen E.M., Christensen P., Gulati S., Unsgård G., Kvistad K.A., Solheim O. (2014). Surgically acquired deficits and diffusion weighted MRI changes after glioma resection—A matched case-control study with blinded neuroradiological assessment. PLoS ONE.

[9] Khan R.B., Gutin P.H., Rai S.N., Zhang L., Krol G., DeAngelis L.M. (2006). Use of diffusion weighted MRI in predicting early post-operative outcome of a new neurological deficit after brain tumor resection. Neurosurgery.

[10] Dützmann S., Geßler F., Bink A., Quick J., Franz K., Seifert V., Senft C. (2012). Risk of ischemia in glioma surgery: Comparison of first and repeat procedures. J. Neurooncol..

[11] Strand P.S., Berntsen E.M., Fyllingen E.H., Sagberg L.M., Reinertsen I., Gulati S., Bouget D., Solheim O. (2021). Brain infarctions after glioma surgery: Prevalence, radiological characteristics and risk factors. Acta Neurochir..

[12] Magill S.T., Nguyen M.P., Aghi M.K., Theodosopoulos P.V., Villanueva-Meyer J.E., McDermott M.W. (2021). Postoperative diffusion-weighted imaging and neurological outcome after convexity meningioma resection. J. Neurosurg..

[13] Strand P.S., Sagberg L.M., Gulati S., Solheim O. (2022). Brain infarction following meningioma surgery-incidence, risk factors, and impact on function, seizure risk, and patient-reported quality of life. Neurosurg. Rev..

[14] Geßler F., Dützmann S., Quick J., Tizi K., Voigt M.A., Mutlak H., Vatter H., Seifert V., Senft C. (2015). Is Postoperative Imaging Mandatory after Meningioma Removal? Results of a Prospective Study. PLoS ONE.

[15] Materi J., Mampre D., Ehresman J., Rincon-Torroella J., Chaichana K.L. (2021). Predictors of recurrence and high growth rate of residual meningiomas after subtotal resection. J. Neurosurg..

[16] Schwartz T.H., McDermott M.W. (2020). The Simpson grade: Abandon the scale but preserve the message. J. Neurosurg..

[17] Driver J., Hoffman S.E., Tavakol S., Woodward E., Maury E.A., Bhave V., Greenwald N.F., Nassiri F., Aldape K., Zadeh G. (2022). A molecularly integrated grade for meningioma. Neuro Oncol..

[18] Smith J.S., Cha S., Mayo M.C., McDermott M.W., Parsa A.T., Chang S.M., Dillon W.P., Berger M.S. (2005). Serial diffusion-weighted magnetic resonance imaging in cases of glioma: Distinguishing tumor recurrence from postresection injury. J. Neurosurg..

[19] Oya S., Kim S.H., Sade B., Lee J.H. (2011). The natural history of intracranial meningiomas. J. Neurosurg..

[20] Spiller M., Tenner M.S., Couldwell W.T. (2001). Effect of absorbable topical hemostatic agents on the relaxation time of blood: An in vitro study with implications for postoperative magnetic resonance imaging. J. Neurosurg..

